# Mortality of people with diagnosed dementia in Germany between 2016 and 2021

**DOI:** 10.1186/s13195-026-02229-8

**Published:** 2026-10-15

**Authors:** Kira Baginski, Sandra Landwehr, Manas K. Akmatov, Claudia Kohring, Jens Bohlken, Stefan Teipel, Bernhard Michalowsky, Doreen Müller, Claudia Hornberg, Annika Hoyer, Jakob Holstiege

**Affiliations:** 1 Biostatistics and Medical Biometry, Medical School OWL, Bielefeld University, Universitätsstr. 25, Bielefeld, 33615 Germany; 2 Department of Epidemiology and Health Care Atlas, Central Research Institute of Ambulatory Health Care in Germany (Zi), Berlin, Germany; 3 Institute of Social Medicine, Occupational Health and Public Health, Faculty of Medicine, University of Leipzig, Leipzig, Germany; 4 German Center for Neurodegenerative Diseases (DZNE) Rostock/ Greifswald, Rostock, Germany; 5 Department of Psychosomatic Medicine, University Medicine Rostock, Rostock, Germany; 6 German Center for Neurodegenerative Diseases (DZNE) Rostock/ Greifswald, Patient-reported Outcomes & Health Economics Research, Greifswald, Germany; 7 Department of Health Research Methods, Evidence and Impact, McMaster University, Hamilton, Canada; 8 Department of Environmental Health Sciences, Medical School OWL, Bielefeld University, Bielefeld, Germany

**Keywords:** Dementia, Mortality, Statistical modelling, Partial differential equation, Epidemiology, Claims data, Germany

## Abstract

**Background:**

Germany’s aging population, driven by rising life expectancy and declining birth rates, has resulted in an increasing number of elderly individuals and various health-related challenges, with dementia being one of the most prevalent chronic diseases. Dementia is an umbrella term encompassing various medical conditions that cause progressive cognitive decline. It is linked to a substantially higher risk of death. However, data on the mortality of dementia in Germany are limited. Therefore, this study aims to estimate the all-cause mortality and mortality rate ratio (MRR) of individuals diagnosed with dementia between 2016 and 2021.

**Methods:**

The age- and sex-specific mortality of people with dementia aged 65 and older, as well as the MRR between the years 2016 and 2021, were estimated using a partial differential equation. The estimation relied on the incidence and prevalence of dementia diagnoses among individuals covered by statutory health insurance and on mortality in the general German population, sourced from the German Federal Statistical Office.

**Results:**

The standardized overall mortality of individuals diagnosed with dementia increased slightly from 18,845 to 20,238 per 100,000, reflecting a rise of 7.4% between 2016 and 2021, with males typically showing higher rates. Overall, mortality increases exponentially with age. However, the age-related mortality rates remained relatively stable from 2016 to 2021, with only a slight upward trend. Individuals with dementia had a higher risk of death compared to individuals without dementia. Females diagnosed with dementia had up to 11 times higher risk of death compared to those not diagnosed with dementia at the same age, while for males it was up to 9 times higher. This difference was most pronounced in the 65- to 70-year age group, but diminished with increasing age.

**Conclusions:**

A dementia diagnosis is associated with increased mortality across all age groups, particularly among younger individuals, although the relative risk declines with age as dementia becomes one of several comorbidities. Targeted interventions to prevent dementia, delay onset, and prolong milder stages remain essential to reduce mortality of people with dementia.

**Supplementary Information:**

The online version contains supplementary material available at https://doi.org/10.1186/s13195-026-02229-8.

## Background

Germany is experiencing population aging due to rising life expectancy and declining birth rates. Over the past three decades, the average life expectancy at birth for males has increased by 5.7 years, from 72.5 to 78.2 years, while females have experienced an increase of 4 years, from 79.0 to 83.0 years [1]. This development has led to a considerable increase in the number of individuals living to advanced ages, posing various health challenges. Among the most frequently encountered age-related conditions is dementia [2]. Approximately 1.8 million individuals with dementia were living in Germany in 2023, and according to estimates, up to 2.7 million people aged 65 and older could be affected in Germany by 2050 [3]. However, recent publications suggest that dementia risk may be decreasing in Germany and other developed countries, which may result in a less steep rise in dementia cases than predicted [4, 5].

Dementia is characterized by progressive cognitive decline and represents a set of symptoms that can stem from multiple causes. Although the rate of progression and the specific symptoms vary among individuals, progressive dependency on a third party is a key characteristic of all dementia types. The most prevalent types of dementia are Alzheimer’s dementia, accounting for 50% to 70% of cases, and vascular dementia, which represents about 15% [2]. With the progression of dementia, individuals increasingly require assistance from others, leading to a point where admission to a long-term care facility often becomes unavoidable. Additionally, dementia is linked to a notably higher risk of death, making it one of the most common causes of death around the world [6, 7]. Recent research from the Netherlands shows that the average time between a documented dementia diagnosis and subsequent institutionalization is about 4 years, and survival following a dementia diagnosis until death is up to 5 years and more [8].

Multiple factors have been documented as predictors of mortality in individuals diagnosed with dementia. These factors range from demographic characteristics, such as age, sex, education, and ethnicity, to disease-specific characteristics, including severity, functional impairment, and specific symptoms [6]. Given that dementia predominantly affects older adults with several coexisting diseases, mortality in these patients often results from complications or secondary conditions arising from the interplay of several underlying health issues. While numerous conditions may be present at the time of death, they may not necessarily represent the immediate cause. As a result, accurately identifying the cause of death in individuals with dementia poses a clinical challenge [7, 9]. These circumstances pose challenges when identifying and documenting causes of death in a comparable manner, which, in turn, leads to limited informative value and interpretation of death cause rankings both within and across countries [10].

Although certain treatments may help in slowing disease progression and improving its symptoms, it is still irreversible, and no proven cures exist so far [11]. Accurate epidemiological data, such as mortality, prevalence, and incidence, are therefore essential for monitoring trends in dementia morbidity over time and for informing patients, health providers, and health policymakers. These estimates are also crucial for health and social care planners, as they help plan suitable services to manage this progressive, currently incurable condition.

A recent claims-based study has found that, despite an aging population, both the incidence and prevalence of diagnosed dementia documented in German primary care practices declined by 26% and 18%, respectively [5]. However, the underlying reasons for this trend remain unclear and can only be hypothesized, underscoring the need for a more thorough investigation to identify the actual contributing factors.

Given that dementia is a chronic condition characterized by a dynamic interplay among incidence, prevalence, and mortality, we intended to investigate mortality patterns of people with dementia in greater detail. Because accurately determining the cause of death among people with dementia can be clinically challenging, our aim was to estimate age- and sex-specific trends in all-cause mortality rates among individuals with a prior diagnosis of dementia, rather than mortality directly attributable to dementia, between 2016 and 2021, based on epidemiological data on dementia prevalence, incidence, and overall mortality. In addition, age- and sex-specific differences in mortality between individuals with and without dementia have not yet been thoroughly examined in Germany. We therefore further aim to quantify these differences by estimating mortality rate ratios (MRRs) comparing people with diagnosed dementia with those without dementia.

## Methods

### Definition of mortality

Because accurately determining the cause of death of people with dementia can be challenging, we estimate the all-cause mortality of people with a documented diagnosis of dementia instead of people where dementia is recorded as the underlying cause of death. This approach accounts for the multifactorial nature of mortality among people with dementia, which may result from complications of dementia as well as from coexisting or other underlying health conditions. Accordingly, throughout the manuscript, we use the term “mortality of people with dementia” to refer to the all-cause mortality occurring among individuals with a diagnosed dementia, rather than mortality due to dementia.

### Population and data

To estimate the mortality and MRR of people with dementia, we used annual prevalence and incidence data on diagnosed dementia from claims data of the German statutory health insurance (SHI) population, comprising approximately 73 million people in 2022—about 88% of Germany’s total population [5]. These estimates were combined with general-population mortality data from 2015 to 2022.

Annual age- and sex-specific prevalence and incidence data for individuals aged 65 years and older were provided for the period 2015 to 2022. The annual sex-specific estimates were obtained from Michalowsky et al. [5]. The study population for each reporting year included insured individuals in the age group ≥ 65 years who utilized SHI-accredited medical services in the reporting year and in the calendar year three years prior, totalling between 15 and 16 million people per year. Individuals considered as having dementia have a confirmed diagnosis of dementia defined according to at least one of the International Classification of Diseases 10th revision (German Modification, ICD-10-GM) codes F00, F01, F02, F03, F05.1, G23.1, G30, G31.0 and G31.82 in at least two quarters within a time period of four consecutive quarters. Individuals who met this case definition at least once in the past three years or in the reporting year were considered prevalent. Individuals who met the case definition for the first time in the reporting year were recorded as newly diagnosed (incident).

**Fig. 1:**
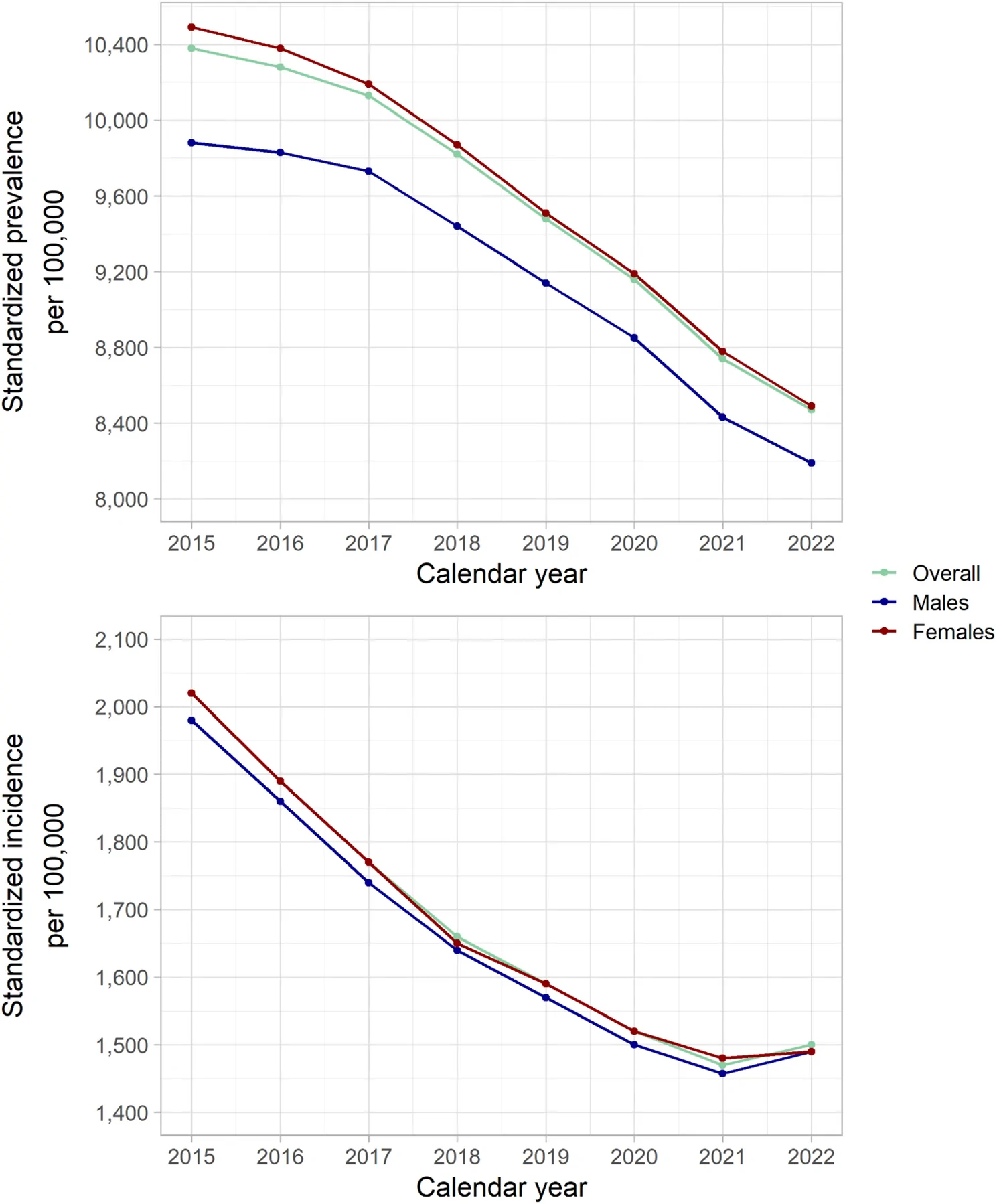
Standardized incidence and prevalence of people with diagnosed dementia per 100,000 individuals covered by statutory health insurance in Germany. Prevalence and incidence were standardized using the age and/or age and sex distribution of the study population and the population at risk in 2015 as the standard populations, respectively [5]

**Fig. 2:**
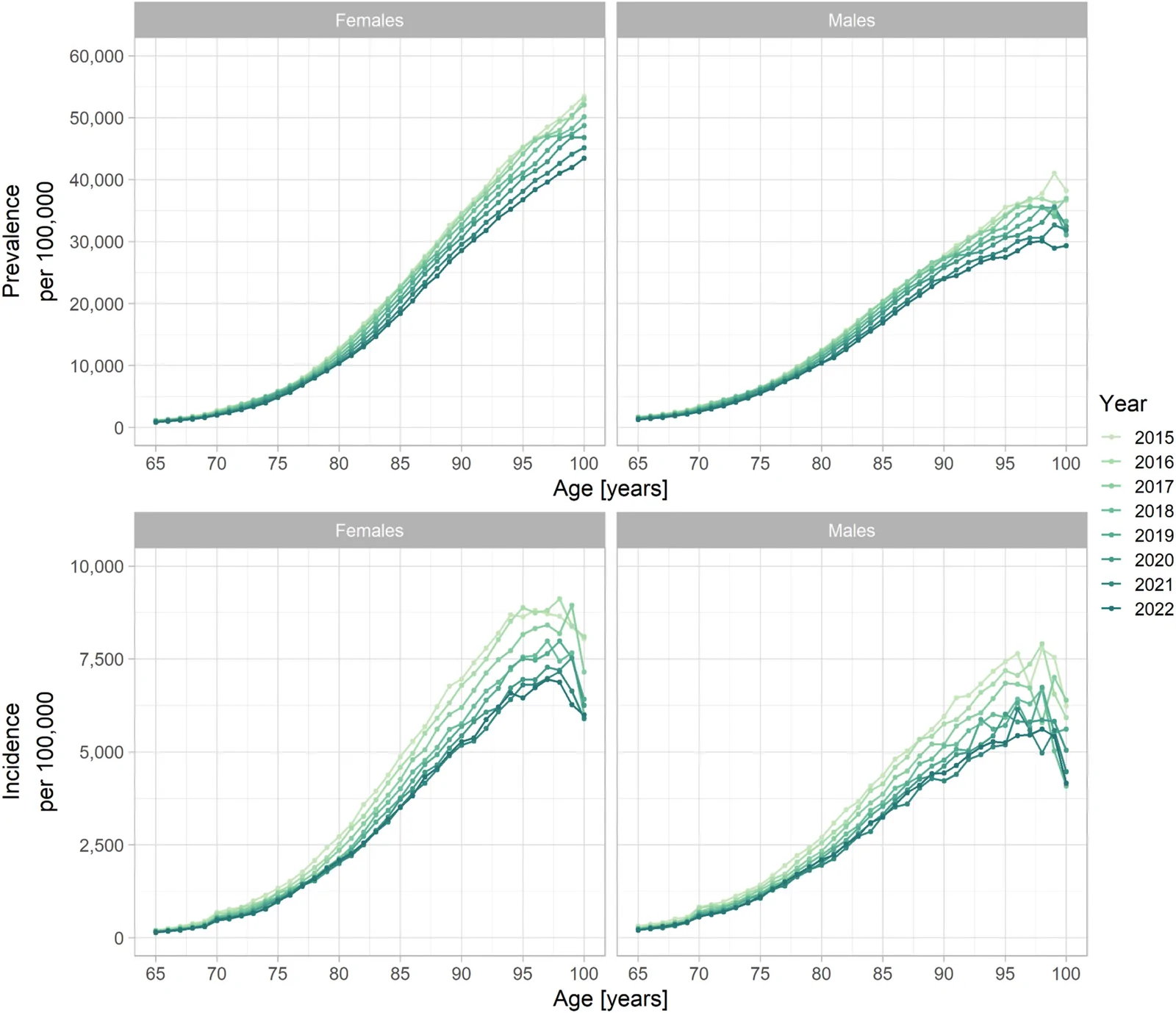
Annual age- and sex-specific prevalence and incidence of diagnosed dementia among the population covered by statutory health insurance in Germany between 2015 and 2022

The data that was used for the annual estimation of the mortality of people with diagnosed dementia that is not specific to age are visualized in Fig. 1. Incidence and prevalence were standardized using the age- and/or age-and-sex distributions of the study population and the population at risk in 2015 as the standard populations [5]. The data used for the annual age- and sex-specific estimation of mortality of people with diagnosed dementia are presented in Fig. 2. Both datasets, including the sex-specific and the age- and sex-specific incidence and prevalence data, were available in aggregated form. For the age- and sex-specific analysis, data were available separately for every single age from 65 to 100 years.

The aggregated mortality data of the general population between 2015 and 2022 were sourced from the German Federal Statistical Office [12]. The corresponding mortality rates were standardized using the age- and/or age-and-sex distribution of the German population in 2015 as the reference population (Supplementary Material Figure S1) [13]. In line with earlier epidemiological research on chronic diseases in Germany, we used the age- and sex-specific mortality rate for the general population based on the 14th coordinated population projections provided by the German Federal Statistical Office (Supplementary Material Figure S2) [14–17].

### Outcomes and statistical analysis

**Fig. 3:**
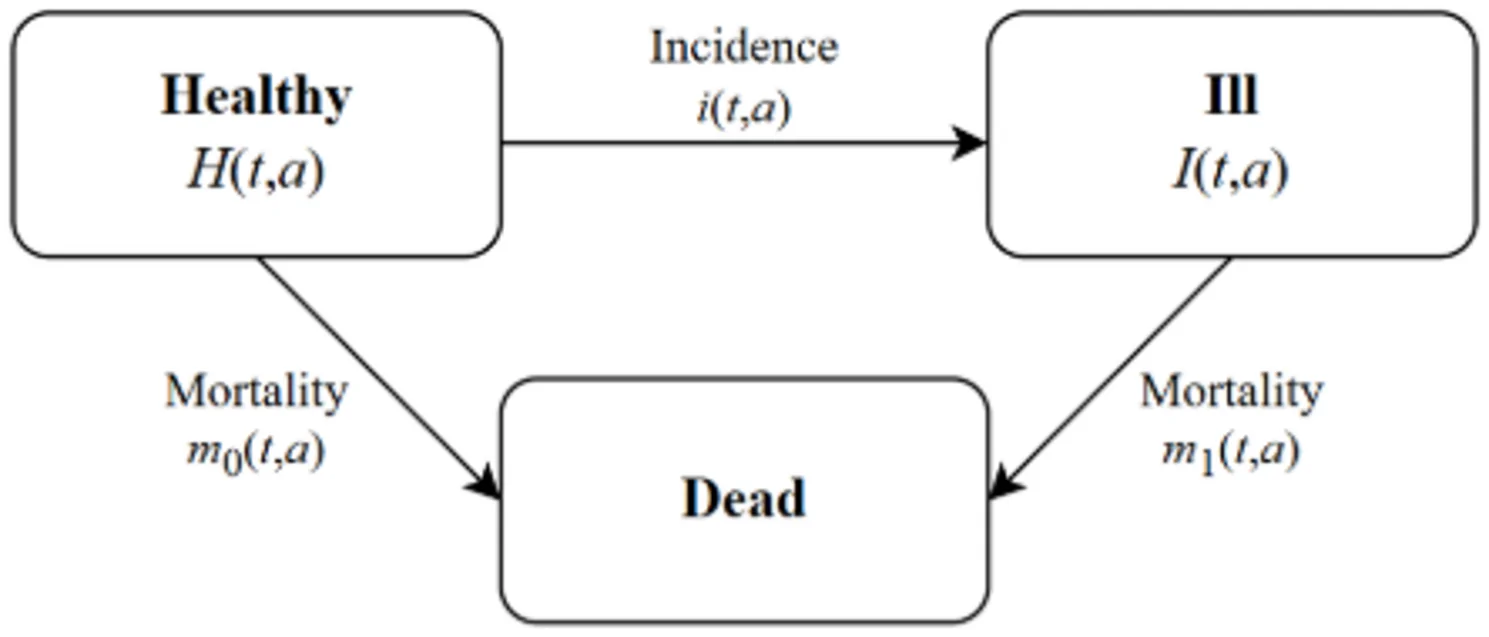
Illness-death model Each individual of a population can be identified as either healthy in relation to the disease of interest, ill, or dead. The transitions among these states are represented by the incidence rate $\:i\:$and the mortality rates for the non-diseased $\:{m}_{0}$ and diseased $\:{m}_{1}$, which depend on both the calendar time$\:\:t$ and the age * a*

To estimate the age- and sex-specific annual mortality rates of individuals diagnosed with dementia and MRRs relative to non-diagnosed individuals, we employed a partial differential equation (PDE) previously applied in the context of chronic disease modelling [15–17]. The foundation for the PDE used in the following analysis is the illness-death model (IDM) depicted in Fig. 3 [18]. The model consists of three distinct states: “healthy” (in relation to the disease of interest), “ill” (representing prevalent cases), and “dead.” It is assumed that every individual within the population belongs to one of these states.

It is a model assumption that all individuals start in the healthy state at birth. Over time, individuals may be diagnosed with the disease of interest, such as dementia, and subsequently enter the ill state, or they may pass away without ever receiving a dementia diagnosis. The rates at which individuals transition between these states are defined by the incidence rate * i* and the mortality rates for the non-diseased $\:{(m}_{0})$ and diseased $\:{(m}_{1})$ individuals. The information regarding states and transitions, along with all estimates in our analysis, is specific to calendar time$\:\:t$ and age * a* [19, 20]. For the sake of clarity, however, the time scales have been omitted from the equations.

Since $\:{m}_{0}$ remains unknown in most diseases, it has been established that the mortality rate of the general population, denoted as * m*, can be included in the PDE. Therefore, the formula $\:m=p\:\cdot\:\:{m}_{1}+\left(1-p\right)\cdot\:{m}_{0}={m}_{0}\cdot[p\cdot\:\left(MRR-1\right)+1]$ can be used to compute the mortality rate of the general population, where * p* indicates the prevalence of dementia and $\:MRR=\:{m}_{1}/{m}_{0}$ is the mortality rate ratio [20].

To calculate the mortality rate $\:{m}_{1}$ and the mortality rate ratio $\:MRR\:$between two time points, it is essential to have the prevalence * p* at those time points, in addition to the general population’s mortality rate * m* and the incidence$\:\:i$ of individuals diagnosed with dementia. The estimation of $\:\:{m}_{1}$ can be conducted by applying the following equation, where $\:\left({\partial\:}_{t}+{\partial\:}_{a}\right)p\:$denotes the temporal changes in prevalence and $\:\partial\:$ represents the differential operator:

$$\:{m}_{1}=\:\frac{i}{p}-i+m-\frac{\left({\partial\:}_{t}+{\partial\:}_{a}\right)p}{p}$$

Similarly, an estimator for $\:MRR\:$is given by the following equation:

$$\:MRR=\:\frac{\left(p-1\right)\cdot\:(\:i\cdot\:p-i-m\cdot\:p+\left({\partial\:}_{t}+{\partial\:}_{a}\right)p)}{p\cdot\:(i\cdot\:p-i-m\cdot\:p+m+\left({\partial\:}_{t}+{\partial\:}_{a}\right)p)}$$

Additionally, the mortality-to-incidence ratio (MIR) was calculated by dividing the standardized all-cause mortality rate of people diagnosed with dementia by the corresponding standardized dementia incidence rate. This measure describes mortality of people with dementia relative to the occurrence of newly recorded dementia diagnoses. All computations were performed using the software R, version 4.4.3. The source code can be publicly accessed on Zenodo [21].

## Results

### Trends in the overall mortality of diagnosed dementia

**Fig. 4:**
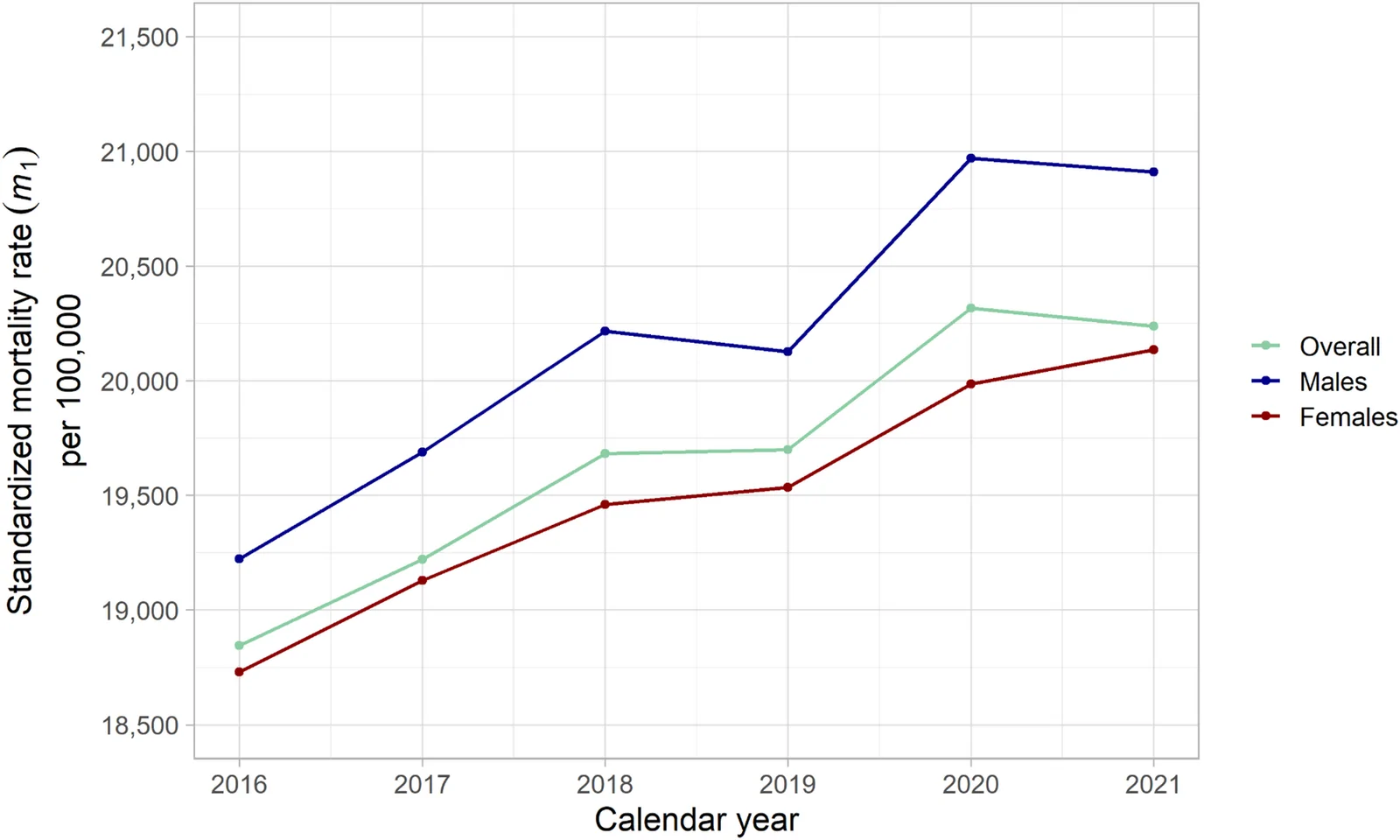
Standardized mortality rate of diagnosed dementia in the German population insured under statutory health insurance

The age-standardized mortality of people with dementia shows an increase from 18,730 per 100,000 in 2015 to 20,135 per 100,000 in 2021 (+ 7.5%) for females and from 19,222 per 100,000 to 20,909 per 100,000 (+ 8.8%) for males (see Fig. 4). Between 2018 and 2019, and between 2020 and 2021, the mortality rate for females shows a comparatively slight increase, while for males it experiences a temporary decrease. The standardized overall mortality of people with dementia demonstrates an increase from 18,845 per 100,000 to 20,238 per 100,000, reflecting a rise of 7.4%. While this rate shows a steady increase from 2016 to 2020, a slight decrease is observed between 2020 and 2021.

Throughout the study period, the MIR was consistently above 10 and showed a continuous increase over time. Across all study years, MIR values were higher in men than in women (see Supplementary Material Figure S3).

### Trends in the age-specific mortality of diagnosed dementia

**Fig. 5:**
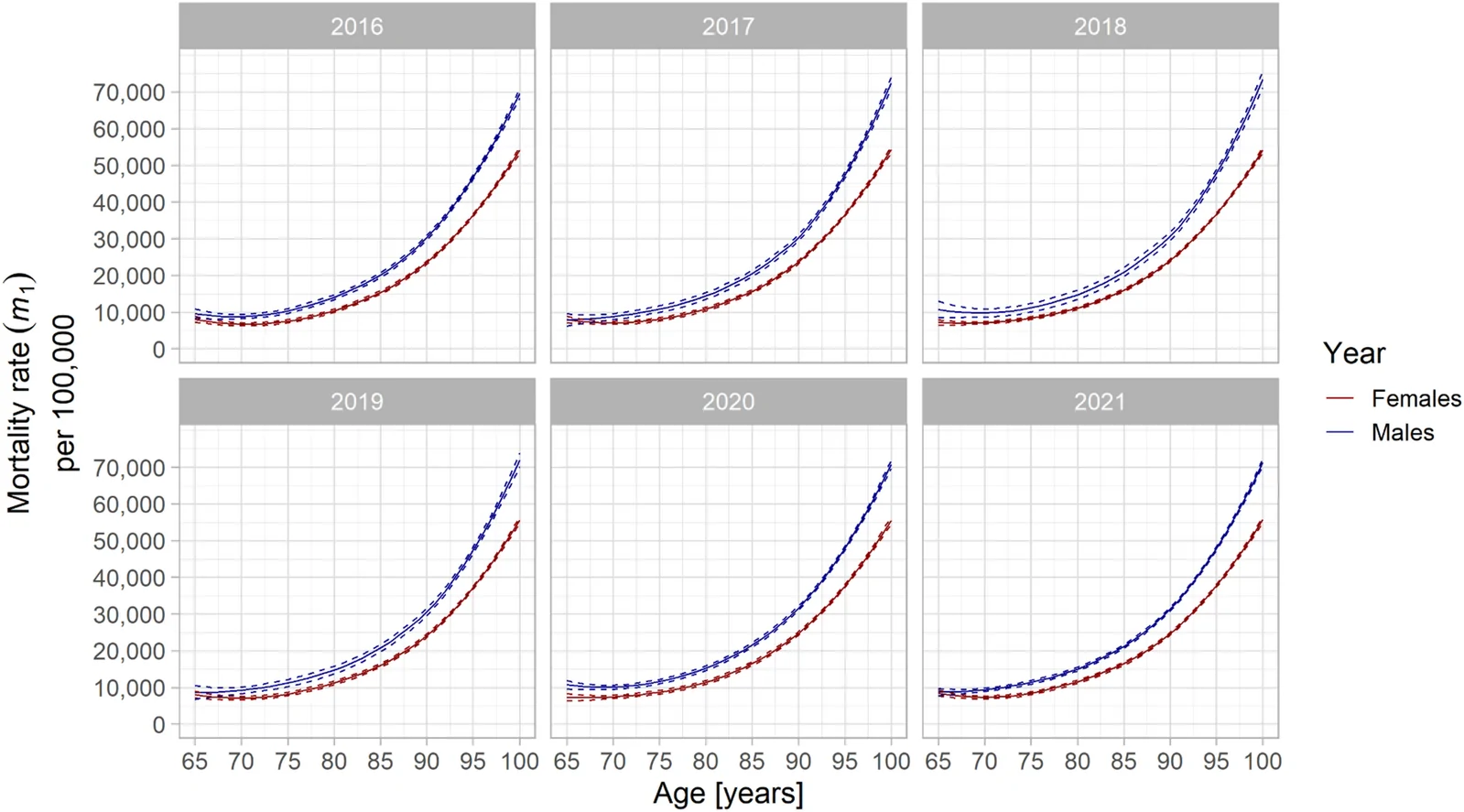
Estimated age- and sex-specific mortality rates per 100,000 with 95% confidence intervals (dashed lines) from 2016 to 2021 of people diagnosed with dementia

The estimated age- and sex-specific mortality rates for individuals diagnosed with dementia are shown in Fig. 5, Table 1 as well as in Supplementary Material Figure S4. Generally, males exhibit higher mortality rates than females. At age 65, the mortality rate of females diagnosed with dementia varies between approximately 7,000 to 8,500 per 100,000 female cases, and for males between 7,000 and 11,000 per 100,000 male cases over the years. By the age of 85, the average mortality rate throughout the years has reached about 16,000 per 100,000 for females and about 21,000 per 100,000 for males. In contrast, among 100+-year-olds, the mortality rate associated with dementia ranges from about 50,000 to 60,000 per 100,000 for females and from roughly 70,000 to 80,000 per 100,000 for males. Overall, mortality rates increase exponentially with age. Nevertheless, the findings indicate that the mortality of people with dementia for both sexes across all age groups has remained relatively comparable from 2016 to 2021, despite a rather upward trend during this period (Supplementary Material Figure S4).

**Table 1:** Trends in age-specific mortality among individuals diagnosed with dementia, shown separately for males and females. Mortality rates are expressed per 100,000 cases within each sex group

	Age	Year						Difference (2016–2021)	
		2016	2017	2018	2019	2020	2021	Absolute	Relative
Males	65	9,587	7,883	10,755	8,621	10,713	8,744	-843	-8.79%
	70	8,816	8,799	9,810	9,232	10,077	9,360	544	6.17%
	75	10,370	10,857	11,203	11,216	11,586	11,399	1,029	9.92%
	80	14,085	14,405	14,757	14,712	15,240	15,021	936	6.65%
	85	20,143	20,546	20,929	20,736	21,523	21,165	1,022	5.07%
	90	30,285	30,307	30,635	30,533	31,623	31,275	990	3.27%
	95	46,778	47,425	47,845	47,373	47,926	47,899	1,121	2.40%
	100+	69,613	72,427	73,452	71,948	70,789	71,311	1,698	2.44%
Females	65	8,045	7,997	7,214	8,031	7,289	8,446	401	4.98%
	70	6,726	7,107	7,143	7,087	7,434	7,302	576	8.56%
	75	7,514	8,055	8,414	8,219	8,719	8,422	908	12.08%
	80	10,384	10,839	11,151	11,204	11,407	11,553	1,169	11.26%
	85	15,382	15,665	16,014	16,040	16,491	16,474	1,092	7.10%
	90	23,541	23,698	24,072	24,088	24,715	24,635	1,094	4.65%
	95	36,433	36,563	36,750	37,213	37,651	37,692	1,259	3.46%
	100+	54,164	54,326	54,163	55,620	55,503	55,743	1,579	2.92%

### Trends in the age-specific mortality rate ratio

**Fig. 6:**
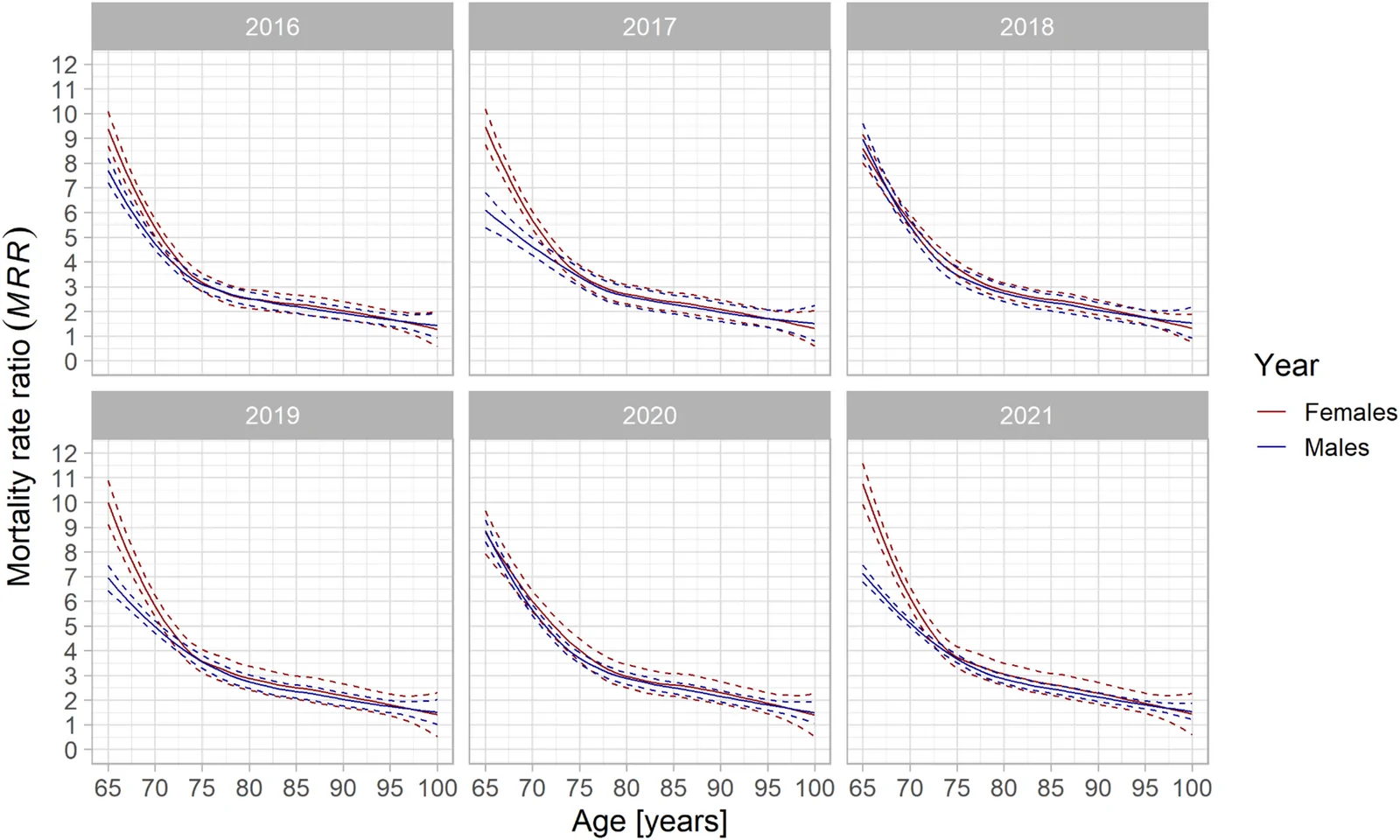
Estimated age- and sex-specific mortality rate ratio with 95% confidence intervals (dashed lines) from 2016 to 2021 between individuals diagnosed with dementia and without dementia

Figure 6 presents age- and sex-specific MRRs with 95% confidence intervals, highlighting a notably elevated risk of death of people with dementia, particularly in the younger age groups under study. At age 65, females with dementia are approximately 8 to 11 times more likely to die than those without dementia, while males with dementia face a 6 to 9 times greater risk of death compared to males without dementia during the period from 2016 to 2021. By age 95, the mortality rates for both males and females with and without dementia show minimal differences over the years. The findings indicate that, from age 75 onward, the MRRs for both sexes increased over the study period. In contrast, among age groups younger than 75, the MRR varies over time, with no clear trend. However, females in this age range show higher MRRs compared to males (Supplementary Material Figure S5 and Table S1).

## Discussion

This study aimed to assess the mortality of people with diagnosed dementia and related MRRs in Germany among individuals aged 65 years and older between 2016 and 2021. Based on aggregated claims and mortality data, the results indicate that the standardized overall mortality for individuals diagnosed with dementia has increased by 7.4% between 2016 and 2021, with mortality being typically higher in males compared to females. The findings also indicate a slight upward trend in the age-and sex-specific mortality of people with diagnosed dementia during this period. Furthermore, individuals with dementia have an elevated risk of mortality compared to those of the same age without dementia, particularly in younger age groups, as the MRR declines with increasing age. The highest risk was observed in the 65–70 age group, where females had a death risk that was up to 11 times higher, while males had a risk up to 9 times higher compared to the same sex in the same age group without dementia.

### Comparison with results from earlier studies

Only a limited number of studies have addressed mortality rates specifically among individuals diagnosed with dementia in Germany, and among those, none have explored age- and sex-specific mortality in such detail. Nonetheless, in general, our findings align with previous reports indicating that mortality among individuals with dementia is higher in males than in females [22–26]. The observed sex disparity is likely due to a range of factors, such as biological differences, hormonal variations, and social determinants [27]. Official statistics on mortality revealed that the rates of mortality due to Alzheimer’s disease and other types of dementia have been on the rise in recent years, comparable to our results [8, 28–30]. Based on data from national registries, a study conducted in Denmark covering the period 2006 to 2015 [26] reported sex-specific mortality rates associated with dementia across different age groups. Their findings reveal a mortality rate of 6.800 per 100,000 for females with dementia and 10.500 per 100,000 for males in the 65 to 69 age range, and 24.600 per 100,000 for females and 35.300 per 100,000 for individuals aged 85 to 89. Our study results for 2016 are very similar: an average mortality of 7.300 per 100,000 for females and 9.100 per 100,000 for males at the age of 65 to 70, and 18.300 per 100,000 for females and 23.800 per 100,000 for males at the age of 85 to 89. Another nationwide registry data study from Denmark, covering 1996 to 2015 [31], and the Swedish Dementia Registry (SveDem) study, covering 2008 to 2011 [25], provide age-specific mortality rates for dementia that are independent of sex. However, the mortality rates for dementia, categorized by sex and/or age, from the three studies fall within a narrow range of our estimates. Variations, whether upward or downward, may be due to differences in the study population, diverse inclusion criteria for dementia patients, and the varying definitions of dementia.

Taudorf et al. [26, 31], and Garcia-Ptacek et al. [25] also evaluated MRRs in people with dementia. However, these MRRs cannot be directly compared to our estimates as they are adjusted for age, year, and/or sex. Nonetheless, the general trend in MRRs across age groups is consistent with our observations. It is also important to note that all three studies pertain to a timeframe preceding ours. More recent estimations are urgently needed, yielding more comparable results.

Our findings underscore that dementia is linked to increased mortality across all affected age groups. The age-related variation in MRRs is likely influenced by the general increase in mortality as a result of population aging. Furthermore, the presence of comorbidities, which are more frequent among the oldest age groups of the population, also plays a notable role in influencing mortality [32]. As a result, when comparing younger dementia patients to others in the same age group from the general population, the latter are likely to have fewer comorbidities and therefore a lower risk of death. However, this difference in mortality tends to diminish progressively with increasing age. Nonetheless, the excess mortality associated with dementia cannot be solely explained by the burden of comorbidities [26]. This may be especially relevant for populations affected at younger ages.

Another factor that may explain the markedly elevated relative mortality risks in the younger age groups relates to diagnostic and care-specific characteristics. In general practice, dementia at these ages is often either undiagnosed or misdiagnosed, and is documented only after a lengthy differential diagnostic process or once it has advanced to a later stage [33, 34]. As a result, routine data predominantly includes moderate to severe cases, while very mild or ambiguous cases are frequently underrepresented. These selection and coding mechanisms may therefore lead to an overestimation of relative mortality risk.

The comparatively pronounced increase in the standardized mortality rates of people with dementia between 2019 and 2020 should be interpreted in the context of both the COVID-19 pandemic and the observed age- and sex-specific patterns. The pandemic may have affected dementia diagnosis and healthcare utilization in 2020, potentially influencing the ascertainment of dementia incidence and prevalence and, consequently, our mortality estimates derived from these measures. Moreover, people with dementia have a high underlying risk of all-cause mortality. COVID-19 may also have caused a temporal displacement of deaths among particularly vulnerable individuals, with some deaths occurring earlier than they would have otherwise. This could have attenuated the observed increase in mortality within a single calendar year. The age-standardized mortality rate was calculated as a weighted average of age-specific mortality rates, using the fixed age distribution of the 2015 standard population. Likewise, the age- and sex-standardized mortality rate was calculated as a weighted average of age- and sex-specific mortality rates, based on the fixed age-and-sex distribution of the 2015 standard population. Therefore, changes in mortality among age groups or age-and-sex groups with higher weights in the standard population had a greater impact on the standardized mortality rates. Therefore, the increase in the standardized mortality rates between 2019 and 2020 may reflect changes concentrated in particular age or age-and-sex groups rather than a comparable increase across all groups. Future analyses accounting for comorbidities, including COVID-19, of people with dementia may further help to explain the mortality patterns observed in this study.

The number of people with a documented dementia diagnosis effectively decreased during the observation period [5]. However, due to population ageing, the number of people with dementia in Germany is expected to increase. This will pose considerable challenges for the health and social care system. To ensure high-quality care, sufficient support, and nursing, therapy, and rehabilitation services must be available for the large number of people affected. At the same time, there is considerable potential for prevention. Although Germany’s overall prevention potential is similar to global estimates, the relevant risk factors differ: because of the high level of education, lower education plays a smaller role, while metabolic syndrome, obesity, and high blood pressure are more important [35].

Multimodal prevention for healthy ageing should therefore be expanded and embedded more firmly in existing care structures, such as general practices, community health centers, workplaces, care facilities, and outpatient services. Support for family caregivers should also be strengthened through low-threshold counseling, self-help groups, and digital platforms. Close cooperation between research, prevention, and care is essential to ensure evidence-based measures are implemented quickly and sustainably [35, 36].

Overall, the estimated mortality rates of people with dementia correspond with the reported decline in the incidence and prevalence of diagnosed dementia cases by Michalowsky et al. [5]. To highlight this, we calculated the MIR (see Supplementary Material Figure S3). In cases of chronic conditions, the MIR may exceed 1 when the number of deaths among existing cases exceeds that of new cases, reflecting declining prevalence. Given that dementia is a chronic and irreversible condition with new cases emerging each year, prevalence can decrease if mortality among those living with the disease exceeds the incidence over calendar time, as observed in our study, reflected by an MIR greater than 1.

### Strengths and limitations

The present study exhibits both strengths and weaknesses. Considering that mortality in dementia patients frequently stems from complications or secondary conditions resulting from the interaction of multiple underlying health-related problems, the estimated mortality refers to individuals who died with dementia, rather than as a direct result of dementia itself. Thorough studies are still necessary to accurately identify the exact cause of death in these cases. Another limitation is that the PDE model assumes that all individuals commence in a healthy state at birth. Although this may not apply to every disease, it is a reasonable assumption within the context of dementia, which leads to an initial prevalence that is effectively zero.

It is important to note that the data used in this study were limited to individuals with dementia who were insured and received services through SHI during a specific timeframe. Moreover, individuals were classified as having dementia only if they had a confirmed diagnosis according to the ICD-10-GM, leaving room for misclassification. It is unknown if the observed decline of documented incident and prevalent dementia cases marks a true epidemiological reduction in dementia burden or if it may be influenced by changes over time in how physicians diagnose dementia in German outpatient care [5]. The true nature of this trend is the topic of ongoing discussions in the German healthcare system and requires further investigation in future primary data studies. While the estimation of incidence and prevalence followed a standardized and rigorously implemented protocol for each year of reporting, changes in the degree of misclassification over time cannot entirely be excluded [5] and may have affected the reported mortality measures. As dementia can be challenging to identify, particularly in its initial phases, where symptoms emerge slowly and differ among individuals, the analysis fails to consider cases of undiagnosed dementia. Furthermore, the findings may not be generalizable to individuals with private health insurance, who make up about 11% of the total German population. Future research is needed to assess whether similar trends exist among privately insured populations. Possible modifications in the delivery of medical care related to dementia, including physician coding and outpatient services, cannot be ruled out.

A key strength of our study is the use of comprehensive outpatient medical claims data comprising all German SHI funds, which encompass the majority of the German population for our analysis. Moreover, both prevalence and incidence are derived from the same country and the identical data-generating process, thus reflecting the same population, except for the population-based mortality, which is obtained from a different data source. Therefore, we were able to determine mortality rates for the population with SHI for every single age from 65 to 100 years. Furthermore, a major strength of our analysis lies in the application of a methodology based on mathematical relationships among prevalence, incidence, and mortality rates. These mathematical connections can help identify trends in disease mortality and assess the effectiveness of preventive strategies. By applying this approach, one can assess mortality among people with dementia, providing important insights for clinical practice and health policy.

## Conclusions

This study highlights that mortality among individuals diagnosed with dementia increased over the study period across all age groups, with the most pronounced rise observed in the younger age groups. Consequently, the risk of death following a dementia diagnosis is particularly high at younger ages. These findings underscore the substantial public health challenge posed by dementia and highlight the urgent need for targeted prevention, early diagnosis, and improved clinical management. Such efforts could be essential in reducing the increased mortality risk associated with dementia, especially in the younger individuals with dementia, and improving the quality of life for individuals affected and their caregivers.

## Supplementary Information

Supplementary Material 1.

## Data Availability

All computations were performed using the software R, version 4.4.3. The data and source code used in this study are publicly available on Zenodo (https://doi.org/10.5281/zenodo.18160932).

## Abbreviations

MRR: Mortality rate ratio
SHI: Statutory health insurance
IDM: Illness-death model
SveDem: Swedish Dementia Registry
MIR: Mortality-to-incidence ratio
PDE: Partial differential equation
