# Supplementary material for: Mortality of people with diagnosed dementia in Germany between 2016 and 2021
Source: Alzheimers Res Ther. 2026 Oct 15;18:233. doi: 10.1186/s13195-026-02229-8 (PMC13673785; doi:10.1186/s13195-026-02229-8)
Supplement: Supplementary file 1 [file 13195_2026_2229_MOESM1_ESM.pdf]

Supplementary Material to

Mortality of people with diagnosed dementia in Germany between 2016 and 2021

Kira Baginski, Sandra Landwehr, Manas K. Akmatov, Claudia Kohring, Jens Bohlken, Stefan Teipel, Bernhard Michalowsky, Doreen Müller, Claudia Hornberg, Annika Hoyer, Jakob Holstiege

Input data

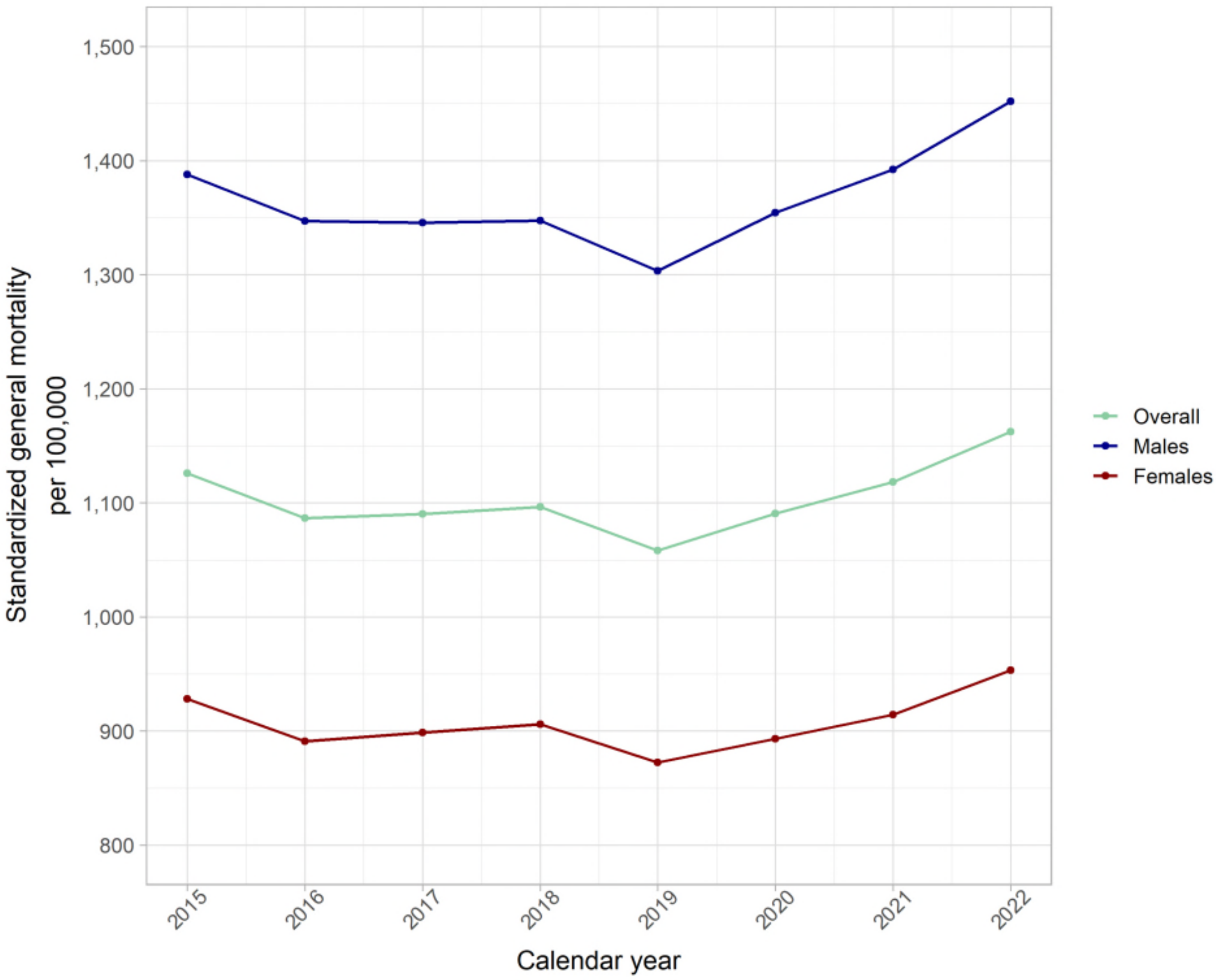

Figure S1 Standardized general mortality of the German population from 2015 to 2022. The mortality rates were standardized using the age and/or age and sex distribution of the German population in 2015 as the reference population [1, 2]

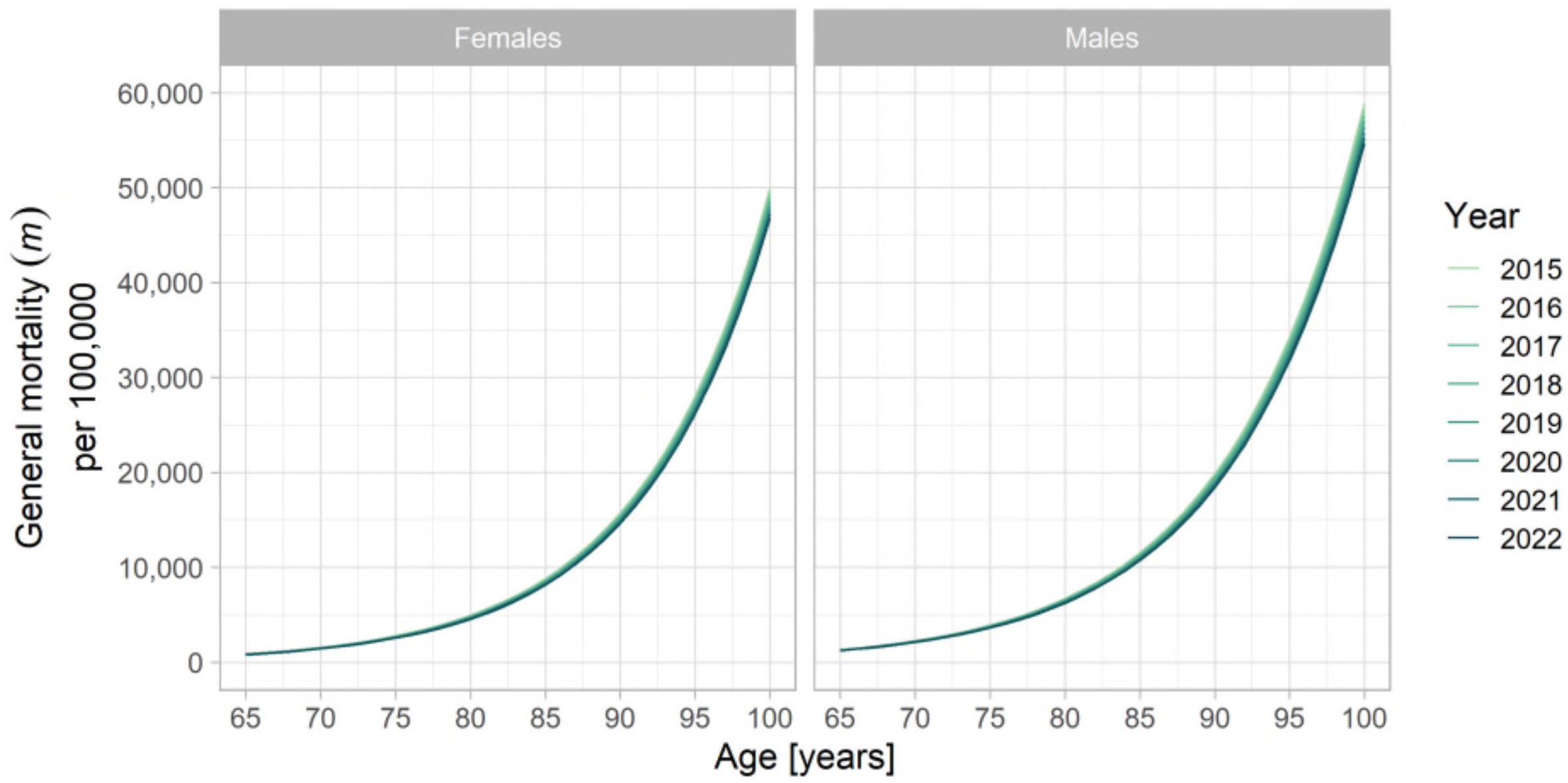

Figure S2 Age- and sex-specific general mortality of the German population from 2015 to 2022 [3]

Results

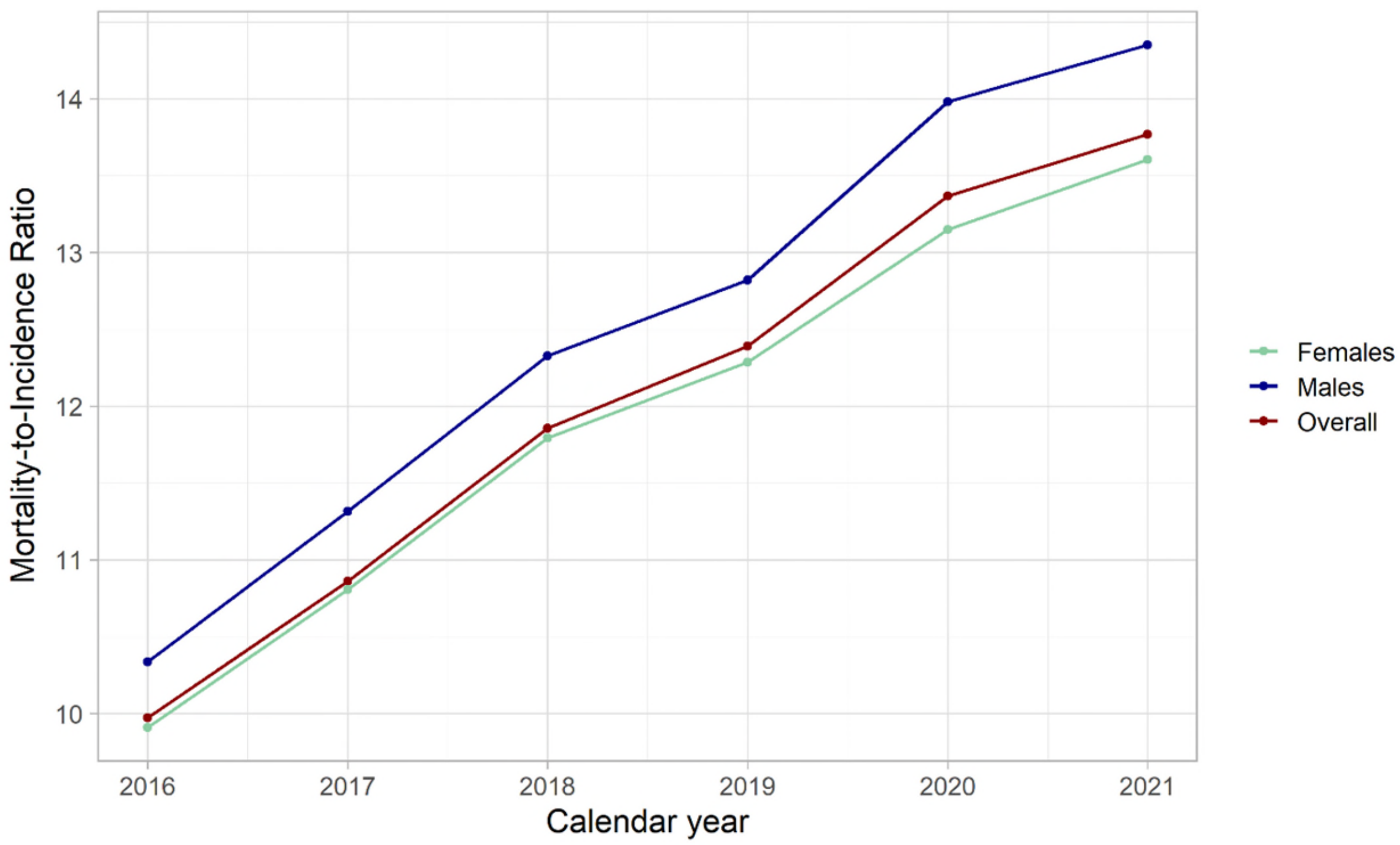

Figure S3 Estimated mortality-to-incidence ratio using age-standardized mortality and incidence rates of dementia

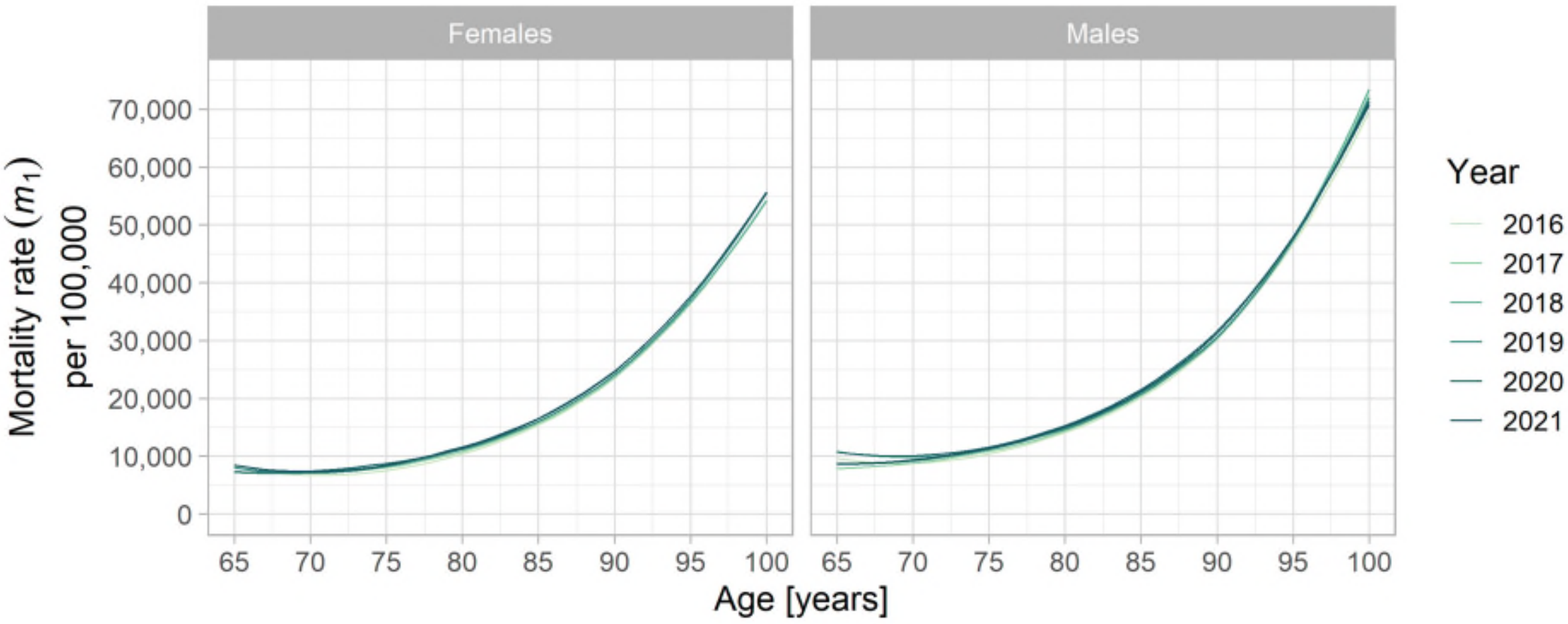

Figure S4 Estimated age- and sex-specific mortality rates per 100,000 from 2016 to 2021 of people diagnosed with dementia

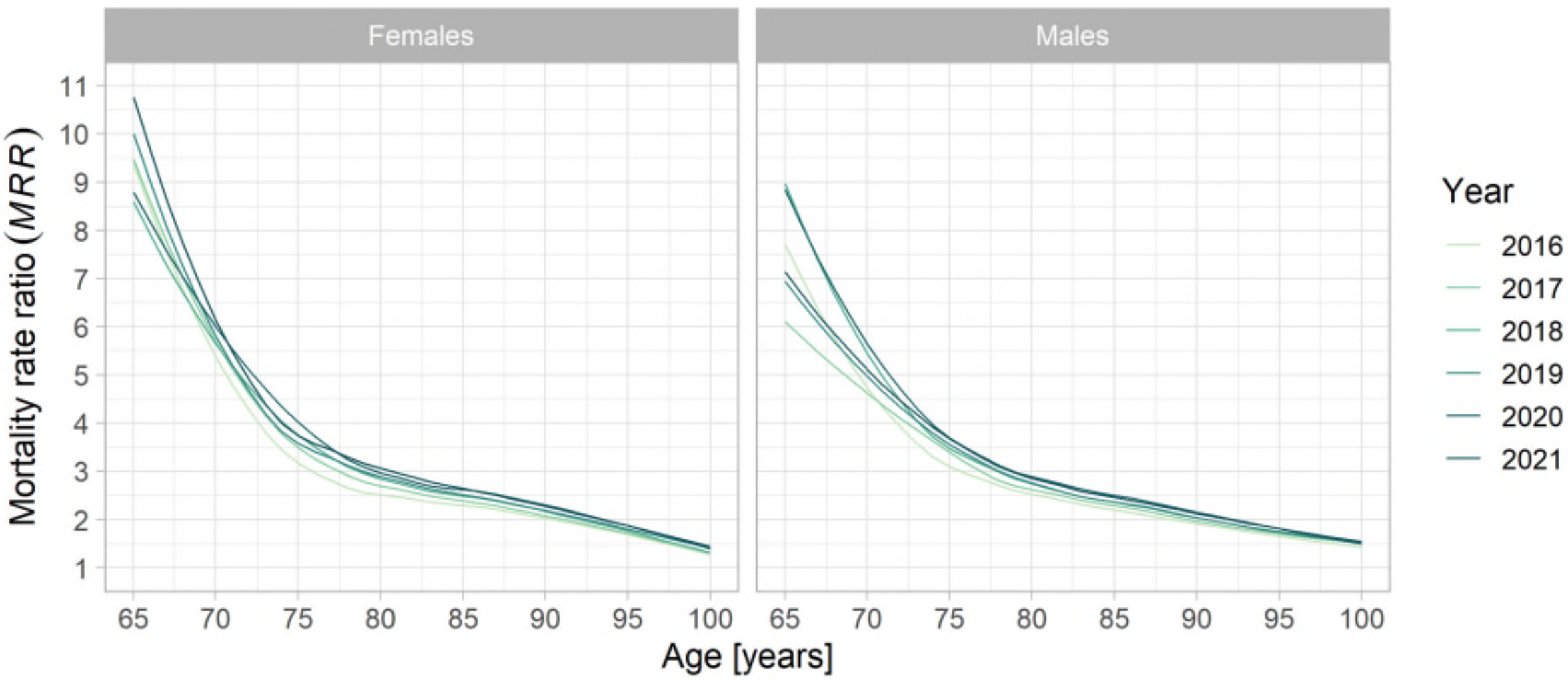

Figure S5 Estimated age- and sex-specific mortality rate ratio from 2016 to 2021 between people with diagnosed dementia and without dementia

Table S1 Trends in age-specific mortality rate ratio, shown separately for males and females

	AGE	YEAR						DIFFERENCE (2016 – 2021)	
		**2016**	**2017**	**2018**	**2019**	**2020**	**2021**	**Absolute**	**Relative**
**MALES**	65	7.71	6.10	8.98	6.94	8.86	7.13	-0.58	-7.52%
	70	4.74	4.62	5.45	4.97	5.64	5.11	0.37	7.81%
	75	3.09	3.39	3.47	3.56	3.69	3.67	0.58	18.77%
	80	2.52	2.61	2.73	2.75	2.88	2.85	0.33	13.10%
	85	2.20	2.28	2.36	2.35	2.50	2.46	0.26	11.82%
	90	1.92	1.97	2.04	2.03	2.15	2.13	0.21	10.94%
	95	1.66	1.70	1.75	1.74	1.81	1.81	0.15	9.04%
	100+	1.43	1.52	1.54	1.52	1.50	1.54	0.11	7.69%
**FEMALES**	65	9.39	9.47	8.59	10.00	8.79	10.76	1.37	14.59%
	70	5.38	5.69	5.65	5.80	6.00	6.15	0.77	14.31%
	75	3.17	3.49	3.75	3.57	4.02	3.74	0.57	17.98%
	80	2.50	2.69	2.84	2.89	2.96	3.05	0.55	22.00%
	85	2.29	2.38	2.49	2.51	2.61	2.64	0.35	15.28%
	90	2.03	2.08	2.17	2.18	2.30	2.28	0.25	12.32%
	95	1.69	1.72	1.76	1.81	1.87	1.87	0.18	10.65%
	100+	1.28	1.31	1.31	1.41	1.40	1.44	0.16	12.50%

References

1. Federal Statistical Office of Germany (Destatis). Deaths: Germany, years, sex, age years. GENESIS-Online database. Accessed October 8, 2025. https://www-genesis.destatis.de/datenbank/online/url/b8044d66
2. Federal Statistical Office of Germany (Destatis). Population: Germany, reference date, age, nationality / sex / marital status. GENESIS-Online database. Accessed October 8, 2025. https://www-genesis.destatis.de/datenbank/online/url/d2de2b87
3. Federal Statistical Office of Germany (Destatis). 14th coordinated population projection from 2019. Accessed April 2, 2025. https://www.destatis.de/EN/Themes/Society-Environment/Population/Population-Projection/_node.html
